# Recurrent neural networks in synthetic cells: a route to autonomous molecular agents?

**DOI:** 10.3389/fbioe.2023.1210334

**Published:** 2023-06-07

**Authors:** Michele Braccini, Ethan Collinson, Andrea Roli, Harold Fellermann, Pasquale Stano

**Affiliations:** ^1^ Department of Computer Science and Engineering, Alma Mater Studiorum Università di Bologna, Campus of Cesena, Cesena, Italy; ^2^ Interdisciplinary Computing and Complex Biosystems Research Group, School of Computing, Newcastle University, Newcastle, United Kingdom; ^3^ European Centre for Living Technology (ECLT), Venice, Italy; ^4^ Department of Biological and Environmental Sciences and Technologies (DiSTeBA), University of Salento, Lecce, Italy

**Keywords:** artificial cells, autonomy, recurrent chemical neural networks, synthetic biology, synthetic cells

## 1 Synthetic biology as an experimental platform to develop chemical AI

The panorama of the so-called Sciences of the Artificial (Cordeschi, 2002) traditionally includes only two approaches: hardware (robotics) and software (AI). Both have been explored, developed and utilized, also in a combined way, to generate useful artifacts such as programmable industrial robots with fixed behavior, various sort of software programs for classification, translation, languaging, management and—more recently—smart robotic devices such as self-guiding cars, interactive robots, or other semi-autonomous systems like robotic lawnmowers. On the other hand, the recent biotechnological arena known as Synthetic Biology (SB) (Endy, 2005) has attracted attention because it provides the scientific and technological bases for the construction of non-trivial artifacts in the bio/chemical domain, that can be exploited in basic and applied research. Not surprisingly, SB has been considered as the third “pillar” of the Sciences of the Artificial (Damiano and Stano, 2018). By putting side by side SB with robotics and AI, two interesting scenarios emerge. Firstly, we recognize the existence of a brand new space for the technological development of artifacts with new capabilities, which are not in the reach of traditional hardware and software approaches, because of the peculiarities of bio/chemical materials that are employed (Grozinger et al.,. 2019). The term “wetware” seems appropriate to describe such an approach, which radically differs from (and is actually complementary to) the other two. Second, by analogy with the AI research trends that aim at modeling the process of thinking, SB paves the way to model *living and cognitive systems* in an unprecedented way, deeply rooted in biological organization (Deplazes-Zemp, 2016; Damiano and Stano, 2023). In other words, SB constitutes a platform for the production of wetware artifacts to be employed in experimental and theoretical investigations, according to a peculiar paradigm: the constructive one, often summarized by the Richard Feynman quote “What I cannot create, I do not understand”.

An intriguing research direction stems from these general considerations. Prompted by recent advancements in SB, here we would highlight the possible utilization of bio/chemical reactivity to generate tools and strategies for a genuinely new AI in the wetware domain. Biologically inspired methods have literally revolutionized AI, think for example, of neural networks, genetic algorithms, and membrane computing—just to mention a few. What if, under the new SB paradigm, similar tools could be realized in the wetware domain? In which respect will these new implementations be different, and why, from software/hardware ones? What sort of behavior can be uniquely generated by chemical AI, and what would be its theoretical (as well as practical) relevance? Can these implementations inspire the design of novel AI systems?

This opinion paper aims at furthering our previous discussions (Stano, 2022a; Stano, 2022b; Gentili and Stano, 2022), and puts the above-mentioned scenario in an Artificial Life perspective (Langton, 1989), highlighting, in particular, a possible contribution for understanding and generating systems with a degree of autonomy—a key property of any living system (Varela, 1979).

## 2 Chemical neural networks inside synthetic cells

**FIGURE 1:**
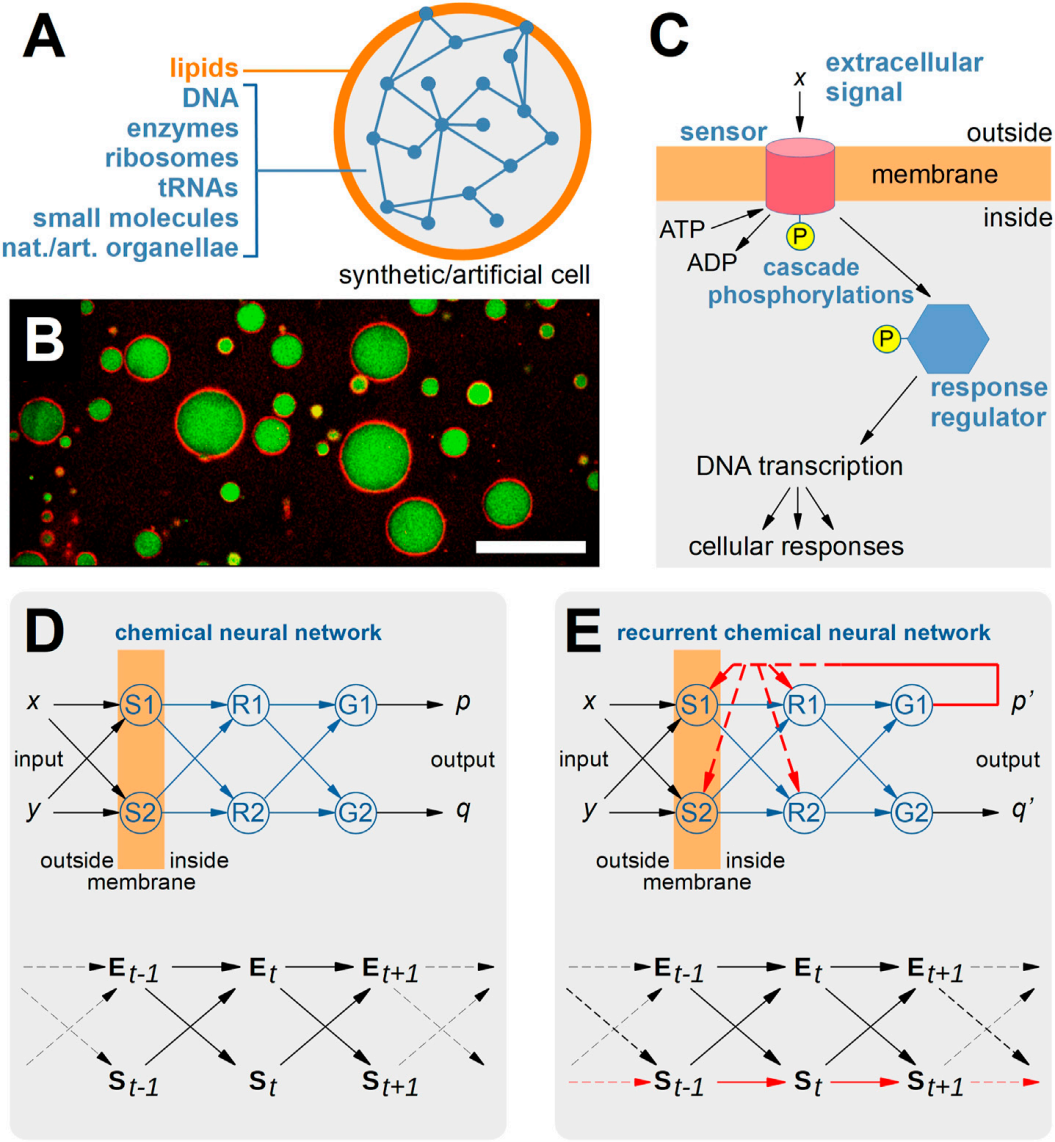
Recurrent Neural Networks in Synthetic Cells. **(A)** Synthetic/artificial cells (SCs) can be constructed via the so-called “bottom-up” synthetic biology approach by several methods that lead to the guided assembly of selected molecular components such as lipids, DNA, ribosomes, enzymes, tRNAs, small molecules, natural or artificial organellae into cell-like structures that roughly resemble the structure and the function of living biological cells. To date, several non-trivial SCs have been built in the lab, ranging from SCs hosting one or more enzyme-catalized pathways, gene expression, lipid synthesis, chemical signaling, etc. **(B)** Typical appearance of SCs (by confocal fluorescence microscopy) producing the green fluorescent protein in their inner volume. The membrane is stained by including a red-fluorescent marker. The size bar represents 25 μm. **(C)** Schematic representation of two-component signaling systems, which enable bacteria to sense, respond, and adapt to their environments, by letting the cell perceive chemical signals present in their surroundings. In a typical system, a membrane protein (*sensor*) with histidine kinase activity catalyzes its auto-phosphorylation in the presence of an extracellular stimulus *x*. Next, the sensor is capable of transferring the phosphoryl group to a *response regulator*, which–thanks to this activation–can then affect cellular physiology by regulating gene expression or by modulating protein activity. **(D)** The mentioned two-component signaling systems can cross-talk (or can be engineered, in principle, in order to enhance cross-talk) so to realize a sort of chemical neural network based on the phosphorylation cascades (called “phospho-neural networks” by (Hellingwerf et al., 1995), as discussed by (Gentili and Stano, 2022; Stano et al., 2022)). In particular, sensors S1 and S2, response regulator R1 and R2, and genes G1 and G2 realize a small chemical neural network with [S1, S2] as input layer, [R1, R2] as hidden layer, and [G1, G2] as output layer. The network performs the computation of extracellular signals (*x*,*y*) into intracellular effects (*p*,*q*). For example, *x* and *y* are small molecules and *p* and *q* are proteins affecting the cellular state. As evidenced by the bottom diagram, the time evolution of the states S of the cell (intended as an “agent”) depends on the states E of the environment. **(E)** The network drawn in **(D)** can be transformed in a *recurrent* chemical neural network if at least one of the outputs is allowed to affect the computation carried out by one of the nodes [S1, S2, R1, R2]. It is possible to imagine several ways this can happen (increase or decrease of sensor and/or response regulator concentration, allosteric regulation by a third-party component). Now, and in contrast with panel **(D)**, the state S of the agent (e.g., S_*t*+1_) will depend not only on the state E_*t*_ of the environment, but also on the state S_*t*_ of the agent. In other words, the agent state *co-determines*, with E, the next agent state. The relative strengths of these two dependencies (e.g., the “weights” of the arrows pointing from E_*t*_ and S_*t*_ to S_*t*+1_) will measure the degree of autonomy of the network (and of the agent). The recurrent CNN can be interpreted as a control module that confers the SCs in which it is embedded a certain degree of autonomy. The bottom diagrams shown in **(D,E)** have been adapted from (Bertschinger et al., 2008).

In previous contributions we have put forward a scenario based on so-called bottom-up “Synthetic Cells” (SCs), which can be fabricated from scratch by a guided-assembly procedure to generate simple solute-filled compartments of the same size and roughly similar structure as biological cells (Figures 1A, B) (Luisi, 2002; Noireaux and Libchaber, 2004; Kita et al., 2008; Lentini et al., 2017; Stano, 2019; Eto et al., 2022). In particular, we have depicted a plausible—but still not realized—design whereby SCs host a biochemical phosphorylation network organized as a neural network (NN), following the seminal discussion provided by (Hellingwerf et al., 1995). In particular, chemical NNs (CNNs) could be realized inside SCs by employing elements of the bacterial two-component signaling systems (Figure 1C) (Gentili and Stano, 2022; Stano et al., 2022).^1^ By analogy with the AI-features of NNs existing in the virtual domain of a software procedure, we refer to CNNs as systems existing in the physical domain that generate a sort of “chemical AI”. The major novelty of CNNs, when compared to NNs, consists in the *embodiment* of the network nodes and links: these network elements are no more logical entities but physical ones, whose behavior is subjected to the physico-chemical laws. Moreover, the “results” of the network computation—i.e., molecules—still belong to the same domain as the network elements, the physical domain. This blurs the difference between “computer and computed”, and allows for interesting “re-entries” of computation products into the computing network, similarly to programs that can modify themselves. Chemical systems have the intrinsic property of being able of self-modification, in the sense that the set of chemical reactions, seen as a whole, can change its parameters (such as binding constants, kinetic constants, fluxes) and connectivity depending on its chemical composition.

There have been several implementations of CNNs using DNA strand displacement reactions that could successfully solve classification problems such as handwriting recognition (Qian et al., 2011). These approaches share in common that they design chemical reaction networks (CRNs) which start in a high energy initial condition that encodes a computational problem. The computation proceeds with the system approaching its thermodynamic *equilibrium*. The basic principle of such CNNs is to tune reaction rate constants and concentrations so that thermodynamic equilibrium concentrations encode the desired computational output (Poole et al., 2022).

Phosphorylation networks can be designed to the same scope (classification problems), whereby the input nodes are sensitive to the presence of chemical signals in the environment, and the gene expression pattern represents the integrated output. Resulting changes in protein expression provides the system specific behaviors. It is possible to imagine a SC endowed with a phosphorylation network which recognizes a certain “environmental pattern” and generates a gene expression pattern—a behavior. Intriguingly, thanks to recent advances in SC technology, we glimpse the opportunity of keeping the system *out-of-equilibrium* by coupling phosphorylation networks with ATP production by separated modules (Lee et al., 2018; Berhanu et al., 2019; Pols et al., 2019; Altamura et al., 2021). It is indeed possible to use a light-induced proton gradient, generated across the membrane of intra-SC organelles, to continuously synthesize ATP from ADP and phosphate. Such an “energizing” module can be coupled to other SC sub-systems (e.g., CNNs), thanks to a continuous flow of energy from light to ATP, and from ATP to energy-requiring reactions.

## 3 Steps toward autonomy?

In order to provide SCs with some degree of agency,^2^ we need to make a step beyond CNNs that address classification problems. In particular, we claim that SCs would benefit from a certain degree of autonomy over their environment. Such a topic is central in Artificial Life because it can guide the production of scientific models for investigating one of the major transitions in the evolution of life, and at the same time it can inspire and support complex processes such as physical computation in an application centered context.

Following the discussion of (Bertschinger et al., 2008), autonomy requires an agent to be only partly determined by its environment, so that it can maintain an internal state or follow an internal program that is, to some extent, not affected by the environment. The agent of course needs to respond to environmental stimuli, but the latter must cope with the internal constraints which ultimately determine what the agent is and how it behaves.^3^ This in turn allows for state dependent computation and for a more complex and meaningful interaction with the environment.

These considerations lead us to propose *recurrent* NNs (Figures 1D, E) as a model to hold state in autonomous agents, and in particular to take them as a theoretical framework to guide future experimental approaches to autonomous SCs. Recurrent networks are characterized by the existence of feedback loops that enable a computation to make use of previously computed outcomes. The input state and internal state computed by the recurrent NNs, at time *t*, both participate in the computation of the internal state at time *t* + ∆*t*. The memory provided by such architectures makes recurrent NNs best suitable in case of sequential events [e.g., in the analysis or generation of time series; a prominent example is that of the so-called Elman nets (Elman, 1990)]. In the context of CNNs, recurrence allows for a “re-entry” of a computed state in the computing mechanism, somewhat realizing the above mentioned organizational closure, yet keeping the agent open to external signals (which, by the way, directly experience the strength of the agent’s internal constraints in order to co-compute the next state). The resulting behavior is thus conditioned both by internal and external states and can give rise to complex interaction with and manipulation of the SCs environment.

The very fact that the dynamics of recurrent network-hosting SCs are constrained by their own output(s), and only co-determined by external factors, further suggests an empirical method to evaluate their degree of autonomy, inspired by Erez Braun’s studies on biological cells (Braun, 2015). The method would consist of placing recurrent CNN-hosting SCs in various experimental conditions, different from each other, aiming at monitoring the possible regularities/patterns of their dynamics—the “stable” part of their behavior in all the different tested scenarios. Clearly, SCs are much less adaptive and plastic than biological cells, but these sort of experiments can be anyway conceived by modifying their environment for example, by varying (perhaps, better conceived as “tuning”) the distribution of input signals in terms of spatiotemporal or chemical patterns (e.g., timing of appearance of signals, concentrations or other extensive quantities, exposure to structural analogues). This scenario is in turn reminiscent of the Kolchinsky-Wolpert operative definition of semantic information (Kolchinsky and Wolpert, 2018; Ruzzante et al., 2023), where “intervened” environment distributions and information flows are ranked based on some crucial properties of the system (e.g., its viability). Semantic information and meaning can be indeed discussed as related to agency and autonomy^3^.

It is worth noting that the demand for recurrence in CNNs puts some constraints on their mathematical and chemical make-up. Because recurrent networks can exploit negative feedback loops to generate non-stationary output patterns from a static environmental input, it can be immediately concluded that these implementations will require input of energy to perform their work. This largely prevents recurrent CNN implementations as closed chemical reaction systems that exploit reversible reactions to rearrange matter towards their equilibrium distribution. The above-mentioned scenario of CNN relying on phosphorylation cascades and gene expression, even in the case of a small number of molecular components, already has the potential to embody out-of-equilibrium conditions. The phosphorylation network ultimately requires ATP, as well as transcription-translation reactions, and a constant degradation of free energy. As mentioned, such a platform can be achieved in current SC technology, where it is possible to conceive a “recharging” photochemical step.

## 4 A scenario deserving exploration

This article just scratches the surface of the question: is it possible to build SCs with a certain degree of autonomy, and how? The impressive technical advancements of the recent years provide the experimental basis for approaching these challenging questions with the confidence that, if not immediate, the scenario can be within reach in the next few years (Buddingh and van Hest, 2017; Salehi-Reyhani et al., 2017; Schwille et al., 2018; Stano, 2019; Gaut and Adamala, 2021; Guindani et al., 2022). Autonomy is at the same time a fundamental feature of biological systems and, in specific cases, a valuable feature of artificial systems. Starting a discussion about how to synthetically achieve it definitely is a timely question, as it can inspire explorative pathways for its modeling and experimental realization.

Here, first of all, we have emphasized that approaching the concept of autonomy from a theoretical perspective requires a departure from the usual linear causality, and calls for a circular—and more systemic—organization and causality. Our initial answer to the question of synthetic production of autonomous systems focuses on recurrent CNNs, intended as autonomy “modules” (or, more modestly—but more realistically—as paths to *minimal* forms of autonomy) that could be engrafted into SCs. Such types of SCs are not easy to implement, but nevertheless the described approach appears as a viable one to provide these systems adaptive capabilities. We claim that the resulting SCs will show (at least traits of) autonomy—a relevant result in the field. Moreover, it will contribute to advancing chemical embodied AI.

As it often happens in explorative research at the intersection of different fields such as AI and SB, open questions refer to the impact of the new approaches in both areas. What can be learned, in SB, from AI? Will these perspectives generate AI-inspired wetware technology? And, *vice versa*, can biology-inspired approaches flow, in an innovative manner, into AI and robotics?

It can be foreseen that advanced SC systems will become new tools for progressing biotechnology in the next decades, and scenarios like smart drug delivery (Leduc et al., 2007), or the so-called Internet of the Bio-Nano Things (IoBNT) (Akyildiz et al., 2015; Kusku and Unluturk, 2021). At a more fundamental level, SC technology can be a platform for crucial investigations of theoretical biology principles. For instance, autonomy can be seen as a prerequisite for agency and other more complex characteristics of living beings. CNNs pose new questions about training, learning and adaptive behaviour. Being capable of the reconstruction of the most well known features typically developed in AI is certainly an appealing goal, and possibly it can hold surprises because of the chemical nature of computing and computed network elements.

## Footnotes

1 Clearly, several technical difficulties must be faced before such an implementation becomes reality. First of all, two-component signaling systems need functional sensors that are transmembrane proteins, notoriously difficult to embed or to produce in SCs. Moreover, fine-tuned cross-talks (Agrawal et al., 2016; Gentili and Stano, 2022) and specific sequestration reactions (Moorman et al., 2019) might be difficult to design. Despite these limitations, the realization of CNN inside SCs appears to be a timely research milestone for the near future.

2 We refer here to the notion of biological agency (Moreno and Mossio, 2015), which includes the capability of a system to generate causal effects. Therefore, biological agents are also autonomous as they are neither completely dependent upon the environment, nor totally independent. Autonomy is then strictly linked to agency and it can be considered a necessary property to achieve it.

3 In the language of Varela, (1979), autonomous agents must accommodate the external variations, which can be seen as perturbations to their own internal dynamics. The agent attributes meaning to external stimuli when they are relevant, both positively and negatively, to its survival (Roli and Kauffman, 2020).
